# Dual modulation of amyloid beta and tau aggregation and dissociation in Alzheimer’s disease: a comprehensive review of the characteristics and therapeutic strategies

**DOI:** 10.1186/s40035-025-00479-4

**Published:** 2025-03-26

**Authors:** Yunkwon Nam, Soo Jung Shin, Vijay Kumar, Jihyeon Won, Sujin Kim, Minho Moon

**Affiliations:** 1 Department of Biochemistry, College of Medicine, Konyang University, 158, Gwanjeodong-ro, Seo-gu, Daejeon, 35365 Republic of Korea; 2 Research Institute for Dementia Science, Konyang University, 158, Gwanjeodong-ro, Seo-gu, Daejeon, 35365 Republic of Korea

**Keywords:** Alzheimer’s disease, Amyloid beta, Tau, Aggregation, Dissociation, Dual-targeting drugs

## Abstract

Alzheimer’s disease (AD) is not a single-cause disease; rather, it is a complex neurodegenerative disease involving multiple pathological pathways influenced by various risk factors. Aggregation and accumulation of amyloid beta (Aβ) and tau are the most prominent features in the brains of AD patients. Aggregated Aβ and tau exert neurotoxic effects in the central nervous system, contributing to the pathogenesis and progression of AD. They also act synergistically to cause neurodegeneration, resulting in memory loss. In this context, dual inhibition of Aβ and tau aggregation, or dissociation of these two aggregates, is considered promising for AD treatment. Recently, dual inhibitors capable of simultaneously targeting the aggregation and dissociation of both Aβ and tau have been investigated. Specific amino acid domains of Aβ and tau associated with their aggregation/dissociation have been identified. Subsequently, therapeutic agents that prevent aggregation or promote disaggregation by targeting these domains have been identified/developed. In this review, we summarize the major domains and properties involved in Aβ and tau aggregation, as well as the therapeutic effects and mechanisms of agents that simultaneously regulate their aggregation and dissociation. This comprehensive review may contribute to the design and discovery of next-generation dual-targeting drugs for Aβ and tau, potentially leading to the development of more effective therapeutic strategies for AD.

## Background

Alzheimer’s disease (AD) is a complex neurodegenerative disease that accounts for the largest proportion of dementia. Over the past century, a variety of AD risk factors, such as sociodemographic, genetic, lifestyle, and environmental factors, as well as physical and mental health conditions, have been extensively studied [1]. Various neuropathological features, including atrophy, cerebrovascular disruption, and proteinopathy, such as amyloid beta (Aβ), tau, transactive response DNA binding protein of 43 kDa, and α-synuclein, have been observed in AD brains [2]. Additionally, the accumulation of Aβ and tau follows several distinct trajectories [3, 4]. However, the most prominent neuropathological characteristics of AD among various types of dementia are the presence of extracellular Aβ plaques and intracellular neurofibrillary tangles (NFTs) composed of hyperphosphorylated tau proteins in the brain [5]. Aβ peptides are produced through the amyloidogenic process, in which amyloid precursor protein (APP) is sequentially cleaved by β-secretase and γ-secretase while bypassing α-secretase [6]. Tau protein becomes abnormally hyperphosphorylated due to an imbalance in enzymes that add or remove phosphate [7]. Accumulation of Aβ peptides and hyperphosphorylated tau occurs ~ 20 years before AD onset [8, 9], influencing various AD-associated pathologies. Aβ also interacts with tau to exacerbate AD progression through multiple synergistic mechanisms [10–14]. In particular, studies in animal models have demonstrated that the Aβ − tau interactions not only accelerate tau pathology but also exacerbate neuronal damage by impairing neurotransmission, disrupting calcium homeostasis, and destabilizing microtubules [15–22]. Interestingly, Aβ pathology is closely associated with the spread of tau pathology [23], especially in its initial regions within the rhinal cortex. In patients with a higher accumulation of Aβ (specifically, an amyloid burden > 40 centiloid), tau pathology is more likely to extend into the neocortex compared to those with lower Aβ deposition [24]. The spread of tau pathology facilitated by Aβ is a better predictor of cognitive performance of AD patients than Aβ pathology [25]. Several studies have proposed the mechanisms of the interactions between Aβ and tau in the AD brain. First, Aβ not only induces hyperphosphorylation of tau but also promotes the spread of tau aggregates to neuritic plaques. This links the spatial and temporal progression of Aβ and tau pathology [11, 17, 26]. Second, Aβ and tau interact through the long-range neural network that connects the neocortex to the entorhinal cortex, an early site of tau neurofibrillary tangle formation, driving initial tau spreading through long-range neural networks [27]. In the late stages of AD, Aβ strongly interacts with tau in the inferior temporal gyrus to accelerate connectivity-based tau propagation to a wide range of neocortical regions [27]. Finally, interactions between Aβ and tau may involve specific pathways, such as FGFR3 signaling which facilitates tau uptake and aggregation [28], and the norepinephrine signaling pathway, in which Aβ oligomers activate glycogen synthase kinase 3β (GSK3β) via the α2A adrenergic receptor, leading to tau hyperphosphorylation [29]. The cascade of events involving the aggregation and accumulation of Aβ and tau represents one of the crucial trajectories in AD pathogenesis. Therefore, it is necessary to understand the mechanisms of Aβ and tau aggregation and to identify promising therapeutic strategies targeting their aggregates.

Interestingly, Aβ and tau exhibit similar biophysical characteristics and aggregation kinetics; most notably, both form aggregates featuring a common β-sheet structure which exerts neurotoxic effects on the central nervous system [30]. These aggregates are typically formed either through primary nucleation, in which monomers aggregate without the contribution of preformed aggregates, or through secondary nucleation, in which preformed aggregates of the same monomer type catalyze the nucleation from monomers [31, 32]. Among the different forms of Aβ and tau, oligomers and protofibrils are more harmful and damaging to neuronal function than the monomeric form [33, 34]. Moreover, Aβ and tau aggregates synergistically interact to maximize their neurotoxicity [35]. In particular, Aβ aggregates interact with tau to produce toxic effects, while phosphorylated tau promotes Aβ-induced damage to mitochondria in healthy neurons [36]. As both the aggregation of Aβ/tau and their interaction play crucial roles in different aspects of AD pathologies, development of drugs that can simultaneously inhibit Aβ and tau aggregation by targeting common structural elements, such as the β-sheet, is of significant interest.

Notably, several studies have explored the potential of dual inhibitors of Aβ and tau aggregation and dual modulators that can dissociate Aβ and tau aggregates [37–42]. In addition, multiple preclinical and clinical trials have investigated the utility of Aβ and tau dual-targeting drugs in the treatment of AD. These include dual Aβ/tau oligomer inhibitors (Takeda Pharmaceuticals and TREVENTIS™), AS-603 (Amyloid Solution), AS-701 (Amyloid Solution), L&J-AD (L & J Bio.), and BEY-2153 (BEYONDBIO; clinical trial No. NCT04476303). BEY-2153 simultaneously targets Aβ and tau by inhibiting the hyperphosphorylation of both tau protein and APP. Interestingly, AS-603 and AS-701, developed by Amyloid Solution, have been reported to target Aβ and tau aggregates by promoting the degradation of both Aβ and tau oligomers and plaques. Preclinical and clinical studies of dual Aβ and tau modulators have shown that a dual regulatory approach to inhibiting Aβ and tau aggregation or dissociating Aβ and tau aggregates may represent an effective strategy for the treatment of AD [37–42].

In this review, we describe the aggregation properties of Aβ and tau and explore aggregation-related sites and mechanisms, focusing on potential targets for dual intervention. In addition, we summarize and discuss recent discoveries on dual-targeting modulators of Aβ and tau, including both aggregation inhibitors and aggregate dissociators, which have been shown to attenuate various AD-related pathologies. Finally, we discuss promising therapeutic strategies that involve targeting the modulation of Aβ and tau aggregation/disaggregation, highlighting their potential in the treatment of AD.

## Characteristics of Aβ and tau aggregation

**Fig. 1:**
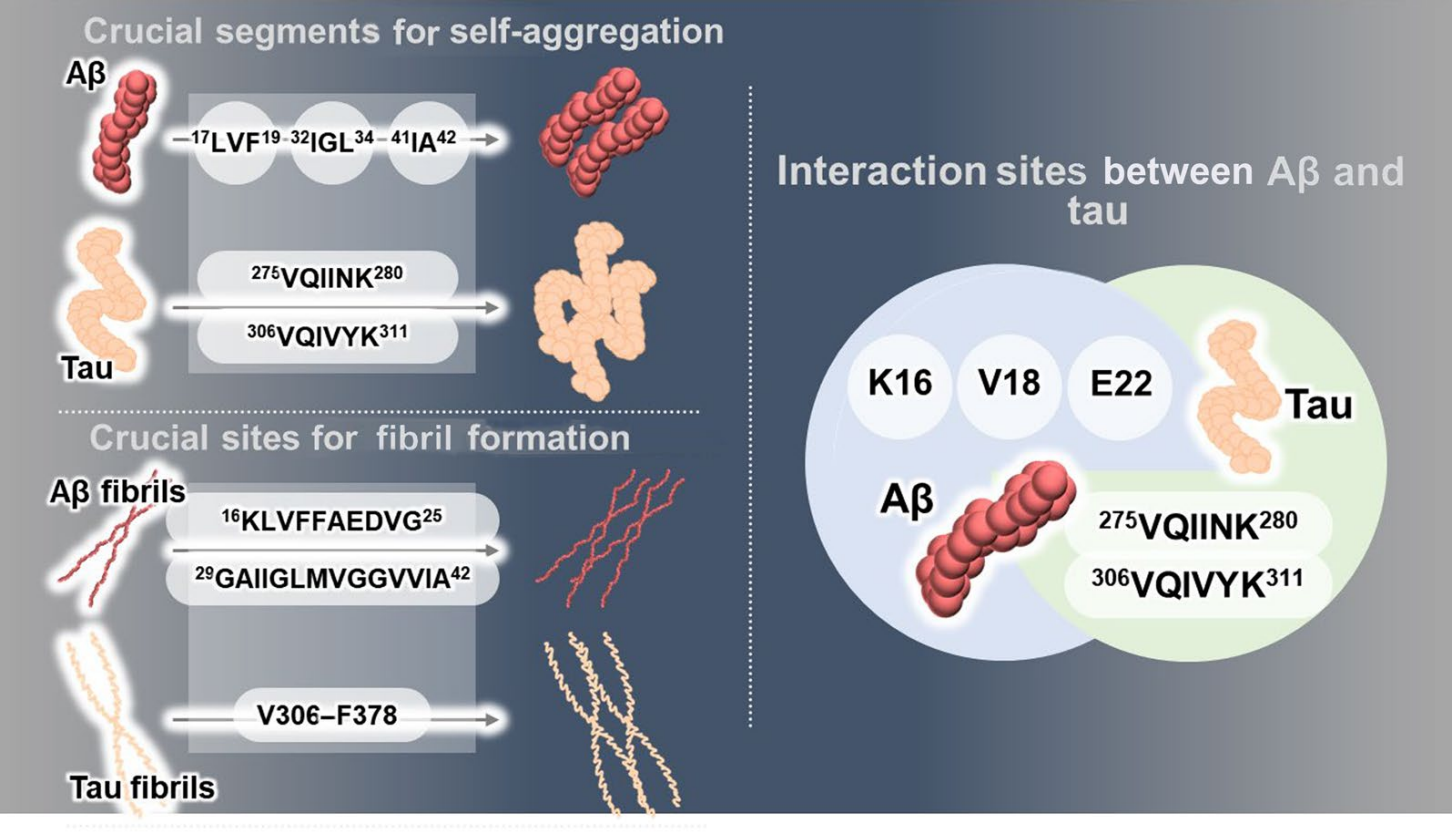
Specific regions involved in Aβ and tau aggregation. Within the Aβ peptide sequence, specific hydrophobic segments, including ^17^LVF^19^, ^32^IGL^34^, and ^41^IA^42^, induce self-aggregation. The ^17^LVFFAED^23^ segment and the ^30^AIIGLMV^36^ segment induce formation of the β-sheet structure of Aβ fibrils. Within the microtubule-binding domain of tau protein, the ^275^VQIINK^280^ and ^306^VQIVYK^311^ segments promote tau filaments to self-aggregate and form helical structures. The cross-seeding process between Aβ and tau is facilitated by specific interactions. In Aβ, the lysine (K) 16, valine (V) 18, and glutamic acid (E) 22 residues are key players in aggregation. For tau, the ^275^VQIINK^280^ and ^306^VQIVYK^311^ segments are crucial for aggregation

Abnormal accumulation of aggregated Aβ and tau is a primary cause of AD. Studies have revealed specific segments in Aβ and tau that are responsible for their aggregation (Fig. 1). Below, we will comprehensively review the mechanisms that lead to the aggregation of Aβ and tau.

### Mechanisms underlying Aβ aggregation and potential therapeutic strategies for modulating Aβ aggregation

Excessive accumulation of Aβ due to an imbalance between Aβ production and clearance is a critical event in the pathogenesis of AD [43]. Interestingly, studies on familial AD associated with *PSEN1* gene mutations have demonstrated a complex pattern of Aβ production: a small number of patients exhibit increased Aβ production, while a large number of patients show decreased Aβ production [44–46]. Surprisingly, a comprehensive analysis of 138 *PSEN1* mutations causing familial AD revealed that only 10 mutations increase Aβ production, while the others either decrease or have no significant effect on Aβ levels [46]. For example, P264L and P267S mutations reduce the processing efficiency of γ-secretase by interfering with PSEN1 self-cleavage, resulting in reduced production of Aβ [44]. Although the overall production is decreased, the production ratio of longer Aβ species such as Aβ_43_ and Aβ_45_ is increased compared to that in healthy controls, whereas the production ratio of shorter Aβ species such as Aβ_38_ and Aβ_40_ is decreased. This finding was unaffected by the characteristics and specific site of the *PSEN1* mutations [45]. Thus, it is increasingly recognized that Aβ accumulation in AD pathology can be caused by impaired Aβ clearance mechanisms even without Aβ overproduction. Aβ initially exists as monomers in a random coil or an α-helical conformation and progressively adopts β-sheet-rich structures during aggregation into oligomers, protofibrils, fibrils, and amyloid plaques [47]. Although certain monomeric forms of Aβ can transiently adopt β-sheet structures under experimental conditions such as multinuclear NMR (nuclear magnetic resonance) spectroscopy, this is not the predominant conformational state of monomeric Aβ. During the aggregation process of Aβ, specific hydrophobic segments of Aβ, such as ^17^LVF^19^, ^32^IGL^34^, and ^41^IA^42^, play crucial roles in promoting self-assembly. In particular, Aβ_42_ exhibits higher hydrophobicity and stronger aggregation properties than Aβ_40_, as it has two additional residues at the C-terminal, isoleucine 41 and alanine 42. This makes Aβ_42_ the more toxic isoform associated with AD [48]. The isoleucine 41 and alanine 42 residues at the C-terminal promote aggregation and amyloid plaque formation by forming hydrophobic interactions with the C-terminal hydrophobic region of Aβ and between different Aβ peptides. Specifically, the C-terminal hydrophobic region of Aβ_42_, the ^32^IGL^34^ segment, competitively interacts with both the hydrophobic central region, the ^17^LVF^19^ segment, and the extreme C-terminal hydrophobic region, the ^41^IA^42^ segment. Notably, interaction between the ^32^IGL^34^ and the ^41^IA^42^ segments results in the exposure of the ^17^LVF^19^ segment to solvent molecules. The exposed ^17^LVF^19^ segment plays an important role in the intermolecular hydrophobic interactions that drive oligomerization of Aβ_42_ [49]. Due to the absence of the two C-terminal amino acids ^41^IA^42^ and the altered competitive interaction between hydrophobic regions, Aβ_40_ exhibits a relatively higher probability of interaction between the ^32^IGL^34^ and ^17^LVF^19^ sections, and, consequently, a lower aggregation tendency than Aβ_42_ [49]. Indeed, it has been well-established that Aβ_42_ aggregates faster than Aβ_40_ due to its higher affinity for self-assembly through hydrophobic interactions [50, 51]. During oligomerization, Aβ preferentially interacts with the ^16^KLVFFAEDVG^25^ segment of other Aβ forms [52]. The ^17^LVFFAED^23^ segment of the central region and the ^30^AIIGLMV^36^ segment of the C-terminal play a crucial role in the formation of the amyloid structure, which is key for the formation of Aβ fibrils [53, 54]. The central and the C-terminal regions form tetramers, each tetramer consisting of two hydrogen-bonded dimers that pack through hydrophobic interactions [55]. Through these aggregation dynamics, Aβ fibrils in the AD brain promote the stacking of Aβ monomers or dimers with similar structures to form amyloid structures, which can further aggregate into fibrils [56–58]. Thus, specific Aβ segments, such as ^16^KLVFFAEDVG^25^ and ^29^GAIIGLMVGGVVIA^42^, significantly contribute to the formation and structural stabilization of Aβ aggregates (Fig. 1).

Considering the aggregation properties of Aβ, diverse compounds can be used to modulate aggregation by targeting specific regions of Aβ. First, Aβ aggregation may be inhibited by an inhibitor binding to the central hydrophobic region of Aβ, the ^17^LVF^19^ segment, to form a hydrophobic interaction. Multiple bioactive components extracted from natural products, including catechins, epicatechin, curcumin, and epigallocatechin gallate (EGCG), have been shown to form a hydrophobic interaction with the central hydrophobic region of Aβ [59–62]. Furthermore, peptide-based drugs with altered structural conformations can interact with the central hydrophobic region of Aβ to reduce aggregation and fibril formation [63–67]. Second, Aβ aggregation may be inhibited through direct interaction with specific regions of the Aβ peptide. For instance, morin and datiscetin target the ^10^YEVHHQ^15^ and ^19^FFA^21^ sequences of the Aβ_42_ monomer, subsequently inhibiting the nucleation and elongation phases of amyloid aggregation, protecting neurons from amyloid toxicity [68]. Quercetin recognizes and binds to the ^17^LVFFA^21^ sequence of the Aβ oligomer during the early stages of Aβ aggregation [69, 70]. In addition, nicotine and its optical isomers exhibit anti-aggregation effects on Aβ through selective interactions with the histidine residues 6, 13, and 14 in the Aβ peptide [71]. One of the cembranoid compounds derived from the Chinese soft coral *Sinularia* sp., exerts an anti-aggregation effect on Aβ not only through hydrogen bonding between the OH at C-11 of this compound and the carbonyl of the backbone of Glycine 37 of Aβ_42_, but also through hydrophobic interactions between the compound and the leucine 34, valine 39, valine 40, isoleucine 41, and alanine 42 residues of Aβ_42_ monomer [72]. Molecular dynamics simulation further demonstrated that both tanshinone I and tanshinone IIA, compounds isolated from the Chinese herbs Danshen or Salvia Miltiorrhiza Bunge, preferentially bind to a hydrophobic amyloid groove formed by the C-terminal sequences ^31^IIGLM^35^ and ^35^MVGGV^39^, along with several aromatic residues [73]. Collectively, these results indicate that the specific targeting and binding of key hydrophobic regions of Aβ is an effective approach to modulating Aβ aggregation.

### Critical mechanisms of tau aggregation and potential therapeutic strategies for modulating tau aggregation

Tau protein progressively aggregates from monomers to oligomers and paired helical filaments (PHFs), eventually forming NFTs [74]. In the AD brain, abnormally hyperphosphorylated tau dissociates from microtubules, destabilizes the normal structural conformation of microtubules, and has increased propensity to aggregate into pathological forms [75]. This process is mediated by several tau kinases including GSK3β and cyclin-dependent kinase 5, and phosphatases [76]. Hyperphosphorylation of tau occurs primarily in the proline-rich regions and the microtubule-binding domain (MTBD), leading to structural destabilization and exposure of hydrophobic regions that promote aggregation of the tau protein [77]. Aggregation of tau is primarily caused by the hydrophobic interactions between MTBDs, which typically contains three or four repeats [78]. The MTBD has specific aggregation-promoting regions that are predominantly involved in the aggregation of tau. Two hexapeptides, ^275^VQIINK^280^ present in the repeat 2 domain (R2), and ^306^VQIVYK^311^ present in the repeat 3 domain (R3) of the MTBD, have been well-characterized as aggregation-promoting regions central to tau aggregation [79, 80]. Valine 306 and isoleucine 308 are central for stabilizing the amyloid structure formed by the ^275^VQIINK^280^ and the ^306^VQIVYK^311^ segments [81]. In particular, the ^275^VQIINK^280^ segment in the R2 domain more strongly contributes to the seeding and aggregation than the ^306^VQIVYK^311^ segment in the R3 domain [79, 82]. However, neither aggregates of hexapeptide ^275^VQIINK^280^ nor aggregates of ^306^VQIVYK^311^ show the helical structure observed in PHFs [79]. These results suggest that the two hexapeptides play a critical role in the initial nucleation of tau protein (Fig. 1). In particular, tyrosine 310 of the ^306^VQIVYK^311^ peptide has been identified as an essential residue for the formation of these tau filaments [83]. In this region, the tyrosine 310 residue engages in a C–H⋯π interaction with isoleucine 308 to form a steric zipper structure in tau filaments, a typical structure for cross-β-sheet forming peptides [84]. Indeed, the structured core of tau filaments extracted from the brains of AD patients contains the ^306^VQIVYK^311^ segment [85]. Tau filaments are composed of two similarly structured protofilaments. The structured core of tau filaments forms a C-shaped architecture comprising eight β-sheets enriched in residues from valine 306 to arginine 406 in the R3 and R4 domains of tau [85, 86]. Importantly, V306–F378, which comprises eight β-sheets, promotes fibrillization and contributes to the stabilization of the fibril structure [87]. Moreover, aggregates of V306–F378 are efficiently internalized through endocytosis, thereby promoting tau propagation [87].

Based on the aggregation properties of tau, many compounds are being developed to modulate tau aggregation to treat AD or tauopathies. First, tau aggregation inhibitors can be covalent; these inhibitors act by forming covalent bonds within residues of the tau protein to prevent aggregation [88]. Methylthioninium chloride, commonly referred to as methylene blue (MB), is a strong inhibitor of tau aggregation [89]. Leuco-methylthioninium bis (LMTX), also known as TRx0237, is a second-generation derivate of MB that binds to abnormal tau and inhibits tau aggregation [90]. MB can modulate tau aggregation by regulating the oxidation of cysteine sulfhydryl groups [91]. In particular, previous studies have shown that the oxidized or reduced forms of MB, known as methylthioninium and LMTX, respectively, bind to the cysteine 291 and cysteine 322 residues of the tau protein, thereby inhibiting tau aggregation [92]. Second, small molecules that interact directly with tau monomers can potentially inhibit the initiation of aggregation by binding to hydrophobic regions. For example, curcumin attenuates tau aggregation through specific molecular interactions. Indeed, structural studies have shown that curcumin binds primarily to the hydrophobic domains of tau, including residues valine 255, valine 292, isoleucine 195, and valine 305. This binding involves not only hydrophobic interactions but also the formation of hydrogen bonds with nearby residues [93]. These hydrophobic interactions and hydrogen bonds both play a critical role in disrupting the steric zipper structure, a key element in tau protein aggregation, thereby inhibiting tau aggregation. Moreover, the rapid change from the exposed state of the hydrophobic region to the normal state reduces the exposure time of the tau hydrophobic residues, thereby reducing the opportunity for interaction with other peptides, subsequently inhibiting aggregation [94]. Finally, tau aggregation may be regulated by blocking the steric zipper structure that forms in cross-β-sheet peptides [88]. In particular, several noncovalent inhibitors of tau aggregation may act by blocking the formation of the steric zipper structures common to cross-β-sheet peptides [88]. Interestingly, some small molecules, including fluorescent dyes, peptide inhibitors D1b, orange G, and curcumin, have been found to disrupt the steric zipper structure across peptides, thus interfering with steric zipper interactions and preventing peptide chains from aggregating into fibrillar structures [88, 95–97]. Taken together, these data suggest that specifically targeting the microtubule-binding repeat domains, especially the aggregation-promoting regions, such as the hexapeptides ^275^VQIINK^280^ and ^306^VQIVYK^311^, through various approaches such as covalent binding, hydrophobic interactions, or disruption of the steric zipper structure, could be an effective strategy to modulate and inhibit tau protein aggregation.

### Critical mechanisms underlying the dual modulation of Aβ/tau aggregation and therapeutic agents

Pathologic Aβ and tau act synergistically to deteriorate neurodegeneration in AD [35]. The cross-seeding of misfolded Aβ and tau, in which the misfolded forms of one molecule catalyze the misfolding and aggregation of the other, accelerates AD progression [98]. The interaction between Aβ and tau is mediated by a variety of non-covalent interactions, including: hydrophobic interactions, electrostatic interactions, and hydrogen bonding (Fig. 1). First, hydrophobic interactions cause induction of Aβ peptide and tau protein aggregation. It has been well established that monomers of Aβ can self-assemble into hydrophobic oligomers and fibrils through hydrophobic interactions [99, 100]. In particular, the hydrophobic interaction sites on Aβ not only induce the aggregation of Aβ, but can also interact with tau to enhance the aggregation of both. For example, the hydrophobic residues of Aβ monomer, including tyrosine 10, phenylalanine 20, methionine 35, valine 39, and isoleucine 41, mainly interact with monomeric tau [101]. Furthermore, residues lysine 16, valine 18, and glutamic acid 22 of the Aβ monomer induce tau aggregation and self-seeding when exposed to solvent molecules [102], indicating the involvement of hydrophobic interactions between Aβ and tau. Second, Aβ aggregates interact with tau monomers through electrostatic interactions. The intrinsically disordered tau protein, when aggregated by pro-amyloid factors, adopts an extended conformation that reduces the structural polymorphism of tau and exposes two hexapeptides, ^275^VQIINK^280^ of R2 and ^306^VQIVYK^311^ of R3 [103]. Aβ aggregates may contain positively charged regions due to the presence of lysine residues or other charged amino acids [104]. The positively charged regions of Aβ aggregates may interact with negatively charged residues, such as glutamine, in the hexapeptide motifs of tau via electrostatic attraction. Moreover, Aβ aggregates or Aβ residues 16–26 bind to the ^275^VQIINK^280^ and ^306^VQIVYK^311^ of tau to form fibrils with a β-sheet axis [105, 106]. Third, the exposure of hydrogen bond donors and acceptors on Aβ aggregates may affect the simultaneous aggregation of Aβ and tau by forming hydrogen bonds with polar residues present in the hexapeptide motifs of tau, such as glutamine and asparagine [101]. Surprisingly, aggregated Aβ, rather than monomeric Aβ, strongly accelerates the aggregation and propagation of tau both in vitro and in vivo [20, 107, 108]. Taken together, these results suggest that the cross-talk between Aβ and tau plays a critical role in AD-associated pathologies, demonstrating the need for therapeutic strategies to disrupt these deleterious interactions.

**Fig. 2:**
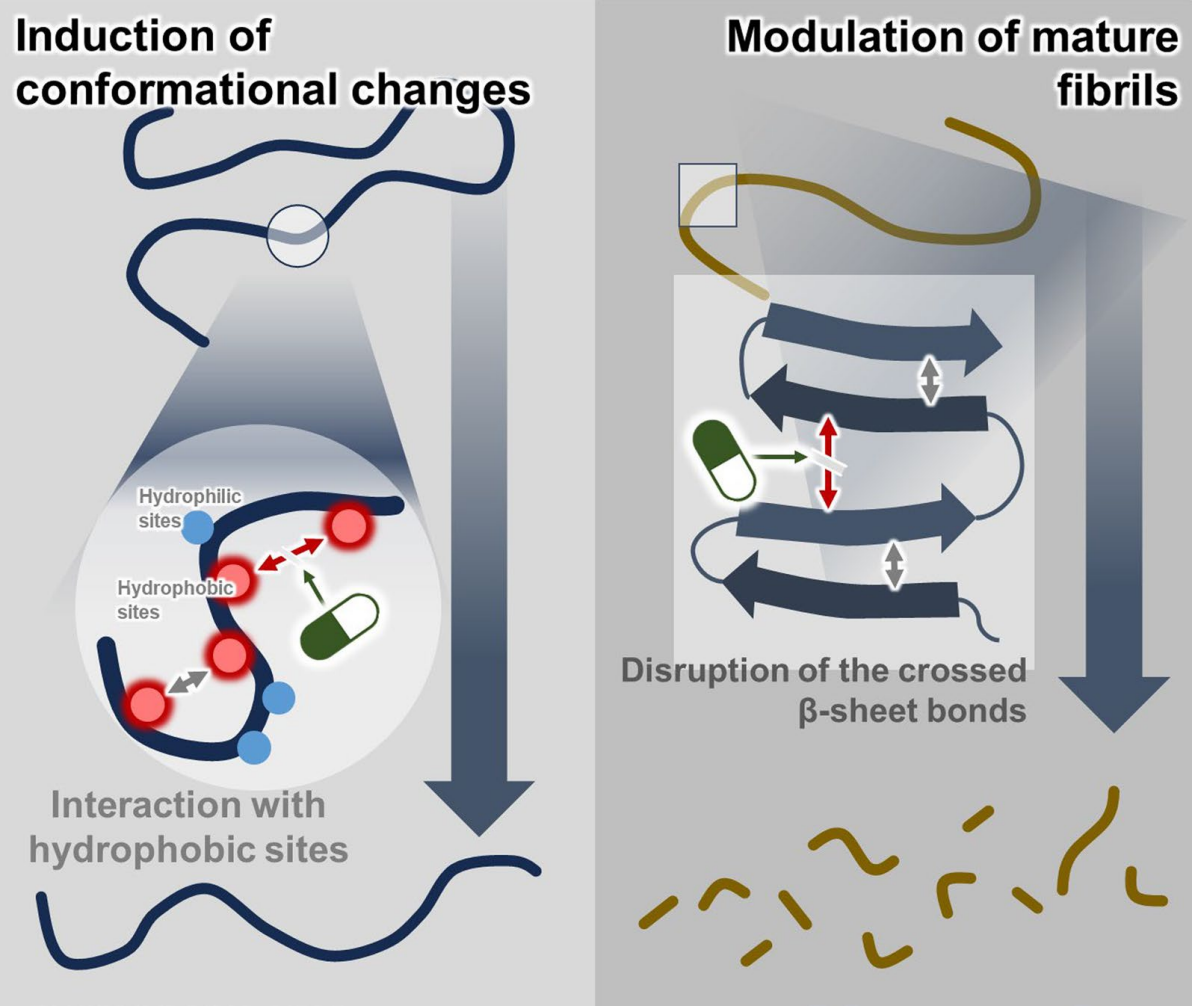
Possible mechanisms of action of dual modulators of Aβ and tau aggregation and their aggregate dissociation. These dual inhibitors of Aβ and tau act (1) to inhibit aggregation by preventing exposure of the hydrophobic regions of Aβ and tau monomers, or by inducing interactions with hydrophobic regions to weaken vulnerable regions of Aβ and tau, and (2) to modulate aggregation by inducing sporadic interactions through covalent or non-covalent bonds with amino acid residues important for Aβ and tau fibrillation. The dual modulators for Aβ and tau aggregate dissociation would induce sporadic interactions through covalent or non-covalent bonds with amino acid residues that are important for the fibrillation of Aβ and tau, leading to dissociation of the fibrils

As described above, simultaneous aggregation of Aβ and tau is primarily driven by hydrophobic interactions, electrostatic interactions, and hydrogen bonds formed by specific residues between them. Hence, dual inhibitors should interact with the key residues involved in the aggregation of Aβ and tau to prevent aggregate formation by disrupting non-covalent interactions, such as hydrophobic interactions, electrostatic interactions, and hydrogen bonding between Aβ and tau [109, 110]. Some agents can regulate Aβ and tau aggregation simultaneously (Table 1). Phenylindane, a primary component of coffee, inhibits the fibrillization of both Aβ and tau [111]. Curcumin has been extensively studied as a simultaneous regulator of Aβ and tau aggregation. Curcumin binds directly to Aβ and tau monomers, stabilizes oligomeric species, and blocks dehydrogenation potentially involved in Aβ and tau interactions [112–115]. Additionally, curcumin derivatives simultaneously inhibit the aggregation of Aβ and tau by blocking hydrophobic interaction and development of the amyloid structure [38, 88, 116]. In addition to curcumin, derivatives of tacrine [39], N-benzylpiperidine [37], benzylamine-hydroxyalkylamine [117], and thiophene [118] have also been reported to exert a dual inhibitory activity on Aβ and tau. These dual inhibitors inhibit the exposure of hydrophobic regions present in Aβ and tau monomers or have hydrophobic/electrostatic interactions with the exposed hydrophobic regions, thereby attenuating aggregation. Furthermore, several agents, including *Uncaria rhynchophylla* [40], genipin and pyrogallol [41], neferine [42], benzylamine-hydroxyalkylamine derivatives [117], necrostatin-1 derivatives [119], curcumin [38, 93, 116, 120], and EGCG [61, 121–124], not only inhibit the aggregation of Aβ and tau but also promote the disaggregation. In particular, genipin and pyrogallol have sporadic interactions through covalent/non-covalent bonds with the amino acid residues that play a critical role in Aβ and tau fibril formation, thereby regulating aggregation or inducing fibril dissociation [41]. Moreover, curcumin, EGCG, and Nec1-derivates inhibit Aβ aggregation and promote the dissociation of Aβ protofibrils by breaking the hydrogen bond [119]. Conversely, EGCG regulates the aggregation of Aβ by forming new π-π interactions with histidine 6 and glutamic acid 11 and forming a hydrogen bond with histidine 14/tyrosine 10 and a hydrogen bond with glutamic acid 11 [124]. EGCG also regulates Aβ aggregation by disrupting the lysine 28–alanine 42 salt bridge through a hydrogen bond with Aβ_42_ as well as the cation-π interaction between its gallic acid ester group and lysine 28 [124]. Furthermore, neferine, curcumin, and EGCG suppress tau aggregation and promote tau dissociation [122]. In particular, EGCG stacks in the polar gap between paired helical filaments. Specifically, EGCG promotes the early disassembly of tau fibrils by competing with the hydrogen bonds, specifically at the serine 341 residue, that maintain tau molecules together in tau fibrils [123]. In addition, neferine, curcumin, and EGCG induce a conformational change from a β-sheet to an unfolded monomeric form, which inhibits aggregation and promotes degradation of Aβ and tau [42, 122]. In this regard, various kinetic studies have demonstrated that the structural disorder of Aβ and tau is crucial for aggregation [125–127]. Unfortunately, among the various interactions that contribute to the anti-aggregation and the degradation effects of dual modulators (such as genipin, pyrogllol, neferine, curcumin, and EGCG) on Aβ and tau, the most important interactions remain unclear. However, the different interactions of the molecules with both Aβ and tau may act in a complementary and synergistic manner. The complementary action may enhance the overall efficacy of dual modulators by targeting multiple interactions involved in Aβ/tau aggregation and disaggregation, providing a more robust mechanism to inhibit or dissociate pathological aggregate formation. Taken together, several approaches could be employed to inhibit Aβ and tau aggregation while promoting their dissociation: (1) inhibiting the aggregation of Aβ and tau by preventing the exposure of hydrophobic regions on Aβ and tau monomers, or through interactions with these regions to weaken areas prone to aggregation; and (2) promoting the disassembly of Aβ and tau by inducing sporadic interactions through covalent or non-covalent bonds with amino acid residues critical for fibrillation (Fig. 2).

**Table 1:** Summary of compounds that inhibit Aβ/tau aggregation and/or dissociate their aggregates

Compounds	Target	Binding regions	Interaction mechanisms	Effects	Models of study	References
Curcumin and curcumin derivatives	Aβ/tau	^17^LVFFA^21^ and ^12^VHHQKLVFF^20^ of Aβ Asp225, Asp194, Lys285, Ser258, Val255, Val292, Leu195, and Val305 of tau	Hydrogen bond, Hydrophobic interaction, Blocking the steric zipper structure, Conformational change that does not favor β-sheet formation	Anti-aggregation effect on tau, Dis-aggregation effect on Aβ and tau	Cell-free system, SAMP8 mice	[38, 93, 116, 120]
Tacrine derivatives	Aβ/tau	N/A	Non-covalent interaction	Anti-aggregation effect on Aβ and tau	HEK-293 T cells	[39]
N-benzylpiperidine derivatives	Aβ/tau	N/A	N/A	Anti-aggregation effect on Aβ and tau	Bacterial cells	[37]
Uncaria rhynchophylla	Aβ/tau	N/A	N/A	Anti-aggregation effect on Aβ and tau, Dis-aggregation effect on Aβ and tau	Cell-free system, 3xTg mice	[40]
Rhynchophylline	Aβ/tau	Glu22, Ala30, Ile31, Asp23, Ala21, Leu34, Val39, Ile41, Phe19, Val40, Ala42, His14, Gly37, and Met35 of Aβ Gln276, Ile278, Val275, Ile277, Val306, Ile308, Gln307, Gly333, His330, Gln336, Lys331 of tau	Hydrogen bond, Hydrophobic interaction, π-π interaction	Anti-aggregation effect on tau, Dis-aggregation effect on Aβ and tau	Cell-free system	
Corynoxeine	Aβ/tau	Ile32, Asp23, Ala21, Leu34, Val36, Val39, Ile41, Phe19, Leu17, Val40, Ala42, His14, Met35 of Aβ Lys274, Ile277, Gln307, Ile308, Gly333, Gln336, Lys331 of tau	Hydrogen bond, Hydrophobic interaction, π-π interaction	Anti-aggregation effect on Aβ and tau, Dis-aggregation effect on Aβ and tau	Cell-free system	
Genipin	Aβ/tau	^15^QKLVFFA^21^ of Aβ ^306^VQIVYK^311^ of tau	Extension of the protein conformation, Hydrophobic interaction	Anti-aggregation effect on Aβ and tau, Dis-aggregation effect on Aβ and tau	Cell-free system, 3xTg mice	[41]
Pyrogallol	Aβ/tau					
Neferine	Aβ/tau	^16^KLVFFAEDVG^25^ and ^29^GAIIGLMVGGVVIA^42^ of Aβ ^275^VQIINK^280^, ^306^VQIVYK^311^,^332^PGGGQ^336^ of tau	Conformational change from β-sheet to α-helix in Aβ	Anti-aggregation effect on Aβ and tau, Dis-aggregation effect on Aβ and tau	Cell-free system	[42]
Thiazolidinedione derivatives	Aβ/tau	N/A	N/A	Anti-aggregation effect on Aβ and tau	Cell-free system, Drosophila	[135]
Benzylamine-hydroxyalkylamine derivatives	Aβ/tau	Lys28, Phe20, and Lys16 of Aβ Gln336 of tau	Hydrophobic interaction	Anti-aggregation effect on Aβ and tau, Dis-aggregation effect on Aβ and tau	Cell-free system	[117]
Phenylindane	Aβ/tau	N/A	N/A	Anti-aggregation effect on Aβ and tau	Cell-free system	[111]
Thiophene molecules	Aβ/tau	Phe19, Ala30 and Ile31 of Aβ Ser36, Ser47, Val34, Leu39, and Ile49 of tau	Hydrogen bond, Hydrophobic interaction, π-π interaction	Anti-aggregation effect on Aβ and tau	Cell-free system	[118]
Epigallocatechin gallate	Aβ/tau	Phe19, Ala30, Gly29, Lys28, Asn27, Glu3, Ala42, Ile41, Glu11, and His13 of Aβ Ser 341 of tau	Extension of the protein conformation	Anti-aggregation effect on Aβ and tau, Dis-aggregation effect on Aβ and tau	Cell-free system	[136]
Necrostatin-1 derivatives	Aβ/tau	^16^KLVFFA^21^ of Aβ	Hydrophobic interaction	Anti-aggregation effect on Aβ and tau, Dis-aggregation effect on Aβ and tau	Cell-free system, 5XFAD mice	[119]

N/A; Not available, Aβ; Amyloid beta, Glu; Glutamic Acid, Ala; Alanine, Ile; Isoleucine, Asp; Aspartic Acid, Leu; Leucine, Val; Valine, Phe; Phenylalanine, His; Histidine, Gly; Glycine, Met; Methionine, Gln; Glutamine, Lys; Lysine, Ser; Serine, SAMP8; senescence-accelerated mouse prone 8

In addition to the dual modulators such as pyrogallol, genipin, neferine, and EGCG, which have known mechanisms for regulating Aβ and tau aggregation, multiple other agents have been proposed to target Aβ and tau aggregation. Carbon dots, a type of carbon-based nanomaterials, have gained significant attention for their therapeutic effects on AD pathology. Interestingly, carbon dots can directly interact with Aβ peptides to inhibit Aβ misfolding and aggregation [128] and prevent tau aggregation [129]. Furthermore, carbon dots derived from congo red have the potential to simultaneously inhibit Aβ and tau aggregation [130]. Molecular tweezers are small molecules designed to bind to specific sites on target proteins, thereby preventing abnormal protein aggregation, such as amyloid-associated deposits [131]. The molecular tweezers can reduce Aβ plaques, NFTs, and microgliosis through modulating Aβ and tau aggregation in 3×Tg mice [132]. Similarly, there has been extensive research on structure-based peptide inhibitors that bind to specific sequences in Aβ and tau [82, 102, 133, 134]. These structure-based inhibitors can suppress the aggregation of Aβ and tau and inhibit their toxicity. Interestingly, congo red is proposed as one of the candidate dual modulators of Aβ and tau aggregation [130]. Congo red is a water-soluble diazo dye commonly used to detect Aβ in AD. It can bind to amyloid aggregates, particularly the β-sheet structure of amyloid fibrils. A study demonstrated that congo red can inhibit Aβ and tau aggregation in vitro, implying a significant potential for the treatment of amyloid-related diseases [130]. Despite numerous studies, the precise molecular mechanisms underlying the effects of carbon dots, molecular tweezers, and congo red on Aβ and tau aggregation have yet to be fully elucidated.

## Advantages and disadvantages of the therapeutic agents

The simultaneous targeting of Aβ and tau offers a multi-faceted therapeutic strategy for the treatment of AD. First, dual targeting of Aβ and tau can prevent the synergistic interaction between them. Studies have shown that Aβ accelerates the aggregation of tau [21, 137] and that tau, in turn, can affect the properties of Aβ plaques [22]. These findings highlight the complex relationship between Aβ and tau, with each molecule exacerbating the aggregation and pathological effects in AD [138]. Moreover, the synergistic effects between Aβ and tau contribute to the exacerbation of AD-related pathologies, including cognitive decline and neurodegeneration [139, 140]. Dual inhibitors targeting both Aβ and tau may not only prevent the formation of Aβ and tau tangles individually but also disrupt the cross-linking, a key mechanism driving the formation of pathological aggregates. Therefore, dual inhibitors targeting both Aβ and tau represent a promising therapeutic approach to mitigating AD pathology by addressing both individual and synergistic mechanisms. Second, multi-target therapeutic approaches can significantly reduce the probability of resistance. Single-target strategies are often limited since proteins can evolve to become resistant to drugs that block specific mechanisms, reducing single-target drug efficacy over time [141]. The simultaneous modulation of Aβ and tau aggregation better reflects the complexity of the biological system and may be more effective in slowing AD progression. Third, dual targeting of Aβ and tau may offer the potential to design therapies specific to a personalized biological profile, in line with the concept of precision medicine. The aggregation patterns of Aβ and tau differ among individuals due to genetic, age-related, and environmental factors [142]. Susceptibility to aggregation varies among patients with AD. For example, one subgroup may have a higher propensity for Aβ aggregation and another subgroup for tau aggregation. Therefore, simultaneous modulation of Aβ and tau represents an innovative approach for AD treatment with the potential to reduce treatment resistance and meanwhile enable personalized treatment.

Despite the promising benefits, there are potential disadvantages to the use of therapeutic agents for inhibiting Aβ/tau aggregation and dissociating their aggregates. The first concern is the specificity and selectivity of Aβ/tau aggregation modulators. The specificity of modulators for Aβ and tau aggregation varies depending on the target regions involved in Aβ and tau aggregation. Aβ modulators target critical hydrophobic regions at which Aβ self-assembles into pathogenic aggregates. Modulators of Aβ also disrupt the sheet-forming segments, interfering with the molecular mechanisms of Aβ aggregation. Similarly, modulators of tau aggregation specifically target repeat domains within the MTBD, characterized by prominent β-sheet-forming and hydrophobic regions that are critical for tau aggregation. However, dual modulators for Aβ and tau aggregation may target and affect common structural elements such as hydrophobic cores and β-sheet regions, which may be present in other proteins, including α-synuclein and prions, and may cause detrimental effects on protein function [143, 144]. Disruption of physiological proteins through non-specific interactions could disrupt cytoskeletal organization, impair synaptic integrity, or dysregulate energy homeostasis, ultimately exacerbating neuronal dysfunction. It can also disrupt protein homeostasis and protein–protein interactions [145, 146]. Dual modulators of Aβ and tau aggregation may provide a safer alternative with fewer side effects compared to anti-amyloid monoclonal antibodies (MABs). While anti-amyloid MABs, such as aducanumab, lecanemab, and donanemab, have demonstrated efficacy in targeting Aβ, they are also associated with a higher risk of amyloid-related imaging abnormalities, brain atrophy, and inflammatory responses, which can result in parenchymal edema and microbleeds [147, 148]. On the other hand, Aβ and tau aggregation modulators have potentially fewer risks and side effects by preventing broad activation of the immune system, compared with anti-amyloid MABs.

The second concern is that dual modulators of Aβ and tau may show heterogeneous therapeutic efficacy in different pathological subtypes of AD. One key challenge is identifying which patients will benefit most from dual inhibitors of Aβ and tau, especially considering that not all AD subtypes exhibit similar levels of Aβ and tau accumulation in the brain. *APOE* ε4 carriers typically exhibit higher Aβ burden in the neocortex and hippocampus and increased tau deposition in the medial and lateral temporal cortices, compared to non-carriers​ [149, 150]. In addition, a study of molecular heterogeneity in AD patients defined at least three different molecular subtypes of AD [151]. As a result of the different subtypes of AD, it may be difficult to predict the clinical efficacy of new inhibitors. However, importantly, current guidelines for AD clinical trials prioritize selecting patients based on biomarkers, such as Aβ and tau [152]. The primary candidates for treatment are those who present with such biomarker profiles, regardless of AD subtype. In addition, both familial and sporadic AD patients undergo accumulation of Aβ, followed by tau, ~ 20 years prior to the onset of AD symptoms [8, 9]. Although it is hard to ensure equal therapeutic efficacy across all subtypes of AD, such a dual-inhibitor approach represents the most promising therapeutic strategy to benefit the widest range of AD subtypes, particularly considering the biomarker-driven patient selection criteria and the fundamental role of both Aβ and tau in the pathogenesis of AD.

The final concern is whether the Aβ/tau aggregation modulators can cross the blood–brain barrier (BBB) and exert therapeutic efficacy for AD pathology. BBB penetration is of considerable concern for treating neurodegenerative diseases such as AD. Unfortunately, numerous tau aggregation modulators identified through in vitro studies have properties, such as permanent cations, that prevent them from crossing the BBB effectively, which limits their efficacy [153]. There is a need for new approaches to improve drug delivery across the BBB which facilitates the transport of Aβ and tau modulators to the brain. First, transferrin-conjugated nanoparticles may have improved delivery of Aβ and tau modulators through receptor-mediated transcytosis [154–156]. Second, nanobodies can cross the BBB through a variety of mechanisms [157, 158] and can therefore be used to deliver conjugated Aβ/tau modulators into the AD brain. Finally, extracellular vesicles and nanomaterials have gained attention as a potential way to facilitate targeted drug delivery across the BBB [159, 160]. These advanced drug delivery strategies can be used to overcome the challenges of BBB permeability, enabling more effective delivery of Aβ/tau modulators and enhancing their therapeutic potential in the treatment of AD.

## Conclusion

Anti-amyloid MABs, including aducanumab (Aduhelm®), lecanemab (Leqembi®) and donanemab (Kisunla®), represent the first generation of disease-modifying therapies for AD and have shown significant clinical benefits in reducing Aβ plaques in the AD brain [161–163]. Targeting Aβ aggregation has proven a clinically relevant approach, advancing AD treatment strategies [164, 165]. Research progress in targeting Aβ aggregation has highlighted the need to address the aggregation of other AD-related pathological molecules, driving the development of therapies aimed at both Aβ and tau aggregation. Here, we review the therapeutic agents that can simultaneously regulate the aggregation of both Aβ and tau in AD, as well as their mechanisms of action. Dual modulators of Aβ and tau aggregation can prevent aggregation and promote disaggregation of Aβ and tau in experimental models of AD. Hydrophobic regions, histidine residues, C-terminal regions, and β-sheet grooves of Aβ are important targets of the modulators. Moreover, the microtubule-binding repeat domains of tau, such as the hexapeptides ^275^VQIINK^280^ and ^306^VQIVYK^311^, are important targets for modulating tau aggregation. In summary, to target Aβ and tau simultaneously, it is important to: (1) prevent the exposure of hydrophobic regions present in Aβ and tau monomers, or induce hydrophobic/electrostatic interactions with the exposed hydrophobic regions, thereby weakening the regions/characteristics involved in aggregation and regulating aggregation; and (2) to induce sporadic interactions through covalent/non-covalent bonds with the amino acid residues involved in the formation of Aβ and tau fibrils, thereby regulating aggregation or inducing fibril dissociation to control aggregation. Nonetheless, although numerous dual inhibitors targeting both Aβ and tau aggregation exist, further research is required to fully understand their binding sites and mechanisms of action. Although inhibition of Aβ peptide and tau protein aggregation has been extensively investigated, therapeutic agents that dissociate their aggregates have been relatively less explored. Nevertheless, it is well known that disruption of the amyloid structure of preformed Aβ and tau aggregates requires a compound with the ability to induce stronger interactions than those that form the β-sheet conformation [166, 167]. However, the discovery of molecules that can mediate sufficiently strong interactions remains a significant challenge.

In conclusion, we review the different sites and mechanisms of action of drugs that regulate Aβ and tau aggregation, suggesting that the development of compounds that dual-target Aβ and tau aggregation based on these mechanisms may be a promising therapeutic strategy for AD.

## Data Availability

Not applicable.

## Abbreviations

AD: Alzheimer’s disease
Aβ: Amyloid beta
APP: Amyloid precursor protein
MAB: Anti-amyloid monoclonal antibody
BBB: Blood-brain barrier
EGCG: Epigallocatechin gallate
GSK3β: Glycogen synthase kinase 3β
LMTX: Leuco-methylthioninium bis
MB: Methylene blue
MTBD: Microtubule-binding domain
NFTs: Neurofibrillary tangles
PHFs: Paired helical filaments
R2: Repeat 2 domain
R3: Repeat 3 domain
